# Highly Flexible and Conductive Printed Graphene for Wireless Wearable Communications Applications

**DOI:** 10.1038/srep18298

**Published:** 2015-12-17

**Authors:** Xianjun Huang, Ting Leng, Mengjian Zhu, Xiao Zhang, JiaCing Chen, KuoHsin Chang, Mohammed Aqeeli, Andre K. Geim, Kostya S. Novoselov, Zhirun Hu

**Affiliations:** 1 School of Electrical and Electronic Engineering, University of Manchester, Manchester, M13 9PL, UK; 2 School of Physics and Astronomy, University of Manchester, Manchester, M13 9PL, UK; 3 BGT Materials Limited, Photon Science Institute, University of Manchester, Manchester, M13 9PL, UK; 4 Manchester Centre for Mesoscience and Nanotechnology, University of Manchester, Manchester, M13 9PL, UK

## Abstract

In this paper, we report highly conductive, highly flexible, light weight and low cost printed graphene for wireless wearable communications applications. As a proof of concept, printed graphene enabled transmission lines and antennas on paper substrates were designed, fabricated and characterized. To explore its potentials in wearable communications applications, mechanically flexible transmission lines and antennas under various bended cases were experimentally studied. The measurement results demonstrate that the printed graphene can be used for RF signal transmitting, radiating and receiving, which represents some of the essential functionalities of RF signal processing in wireless wearable communications systems. Furthermore, the printed graphene can be processed at low temperature so that it is compatible with heat-sensitive flexible materials like papers and textiles. This work brings a step closer to the prospect to implement graphene enabled low cost and environmentally friendly wireless wearable communications systems in the near future.

Wireless wearable communications is a field of increasing research interest due to the numerous potentials offered in areas such as healthcare and fitness monitoring^1^,^2^, mobile network/internet^3^, smart skin^4^,^5^,^6^ and functional clothes^7^ to name a few. Radio frequency (RF) front-end is a basic building block in any communication systems, which transmits and receives RF signals. A RF front-end includes passive components such as antennas, transmission lines (TLs) and impedance matching networks and active circuits such as power amplifier, low-noise amplifier (LNA), frequency mixer and local oscillator^8^ to name a few. Conventionally, a RF front-end is mainly assembled using PCB (printed circuit board) technology, which poses a big challenge in integration with flexible substrates like papers and textiles^4^. To tackle this, coating/plating metal on textile yarns was proposed^9^,^10^. However, in these approaches, even though the metals were deposited on flexible substrates, the fabrication procedures were complicated and low-efficiency, and materials used were expensive, not suitable for mass deployment in low cost wireless wearable applications. Silver nanowires (AgNWs), conductive polymers, carbon nanotubes have also been developed for wearable electronics applications. Although AgNWs is highly conductive^11^, to obtain low enough sheet resistance for RF applications, a relatively thick AgNWs coating is needed^11^,^12^ (230 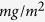 for nearly 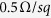
11), which results in high cost for mass production as silver is scarce and expensive^13^. As to conductive polymer, while it can be used for flexible electronics such as sensors, solar cell, its conductivity is too low to be employed for RF signal transmission and radiation^14^,^15^. Conductive polymer is also limited by chemical and thermal instability^16^. Carbon nanotubes, with typical sheet resistance above 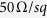, due to high junction resistance between overlapped nanotubes^17^,^18^, is still not conductive enough to meet practical RF circuit requirements.

However, graphene, the allotrope of carbon nanotube, is a very promising material for wireless wearable communications applications owing to its high conductivity and unique properties^5^,^19^. To date, researchers have intensively explored the applications of graphene to make active devices such as transistors and diodes. A quaternary digital modulator was achieved using two graphene transistors^5^. Amplifiers at RF bands were demonstrated experimentally with graphene field-effect transistors^20^,^21^. Other active devices such as frequency mixer^22^,^23^ and oscillator^24^,^25^ were also demonstrated. More recently monolithic graphene RF receiver integrated circuit (IC) performing signal amplification, filtering and down-conversion has also been reported^26^.

However, even though profound progress has been made in graphene active devices, the pace of developing graphene passive RF components has far lagged behind. This is because, in spite of graphene’s high conductivity, both exfoliated and CVD (chemical vapor deposition) graphene sheets have very high surface resistance, hindering their applications in RF passive components^27^,^28^. However, recent development of graphene conductive ink has brought the possibility along with its superiority in high conductivity, mechanical flexibility, light weight and low cost^29^,^30^,^31^. Preparation of graphene conductive inks can be generally categorized into two groups. One is binder-free technique which disperses the graphene directly in solvents such as N-Methyl-2-pyrrolidone or Dimethylformamide (NMP/DMF) without adding any binder^31^,^32^, whereas the other uses binders like ethyl cellulose (EC)^29^,^33^. Even though the latter technique can offer higher conductivity, it requires high-temperature thermal annealing, making it incompatible with heat-sensitive substrates like papers and textiles^18^. On the other hand, binder-free technique is compatible with heat-sensitive substrates thanks to its low temperature annealing^32^, however much further improvement of ink conductivity is required for RF applications.

We have developed binder-free technique which is not only compatible with heat-sensitive substrates like papers and textiles, but also offers high conductivity and mechanical flexibility^34^. The technique is aimed for industrial scale screen printing. The measured conductivity from this technique reaches 4.3 × 10^4^ S/m, which is almost double of 2.5 × 10^4^ S/m from previously reported RGO (reduced graphene oxide) with binder and 10 times higher than that from binder-free method^29^,^32^. In this report, this highly conductive printed graphene is further utilized to construct transmission lines and antennas on a flexible substrate such as paper. The performances of these components, especially under different bending cases, are experimentally examined in communication frequency bands, such as mobile cellular and WiFi spectrums. The results demonstrate that printed graphene enabled RF passive components have desired property and quality for wireless wearable communications applications. Together with the aforementioned progress of graphene active RF devices, a truly all graphene enabled wireless wearable communications system can be expected in the near future.

## Results

### Printed graphene preparation and characterization

**Figure 1:**
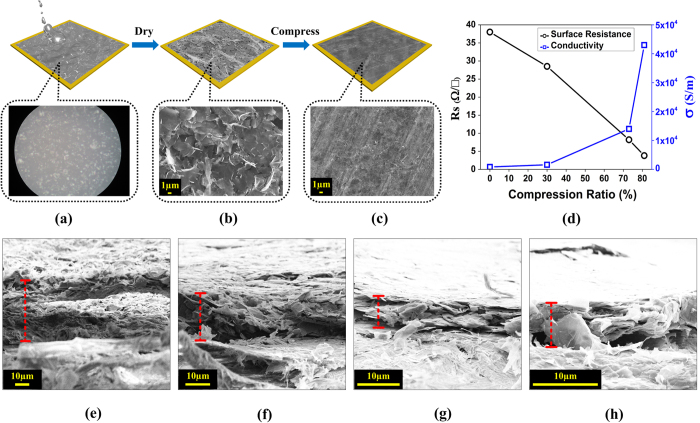
Schematic illustration of preparing printed graphene and its characteristics. (**a**) Graphene nanoflake ink is coated on substrate, (**b**) After drying, highly porous graphene nanoflakes coating forms, (**c**) Highly dense printed graphene is obtained with compression, (**d**) Conductivity and surface resistance under different compression ratios. (**e**) Cross sectional SEM image of uncompressed sample, with thickness around 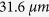. (**f**) Cross sectional SEM image of sample with compression ratio 30%, with average thickness around 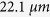. (**g**) Cross sectional SEM image of sample with compression ratio 73%, with thickness around 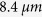. (**h**) Cross sectional SEM image of sample with compression ratio 81%, with thickness around 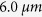.

The RF passive components in this paper are made of printed graphene. Here we briefly introduce the preparation of printed graphene, and the details are included in the Method section^34^. Normally, conductive ink contains binders such as polymeric, epoxy, siloxane, or resin binders because granular powders cannot form a continuous film without linkages of them. However, binders need to be decomposed or evaporated through high-temperature thermal annealing. This high-temperature process prevents graphene ink from printed on flexible substrates such as papers and textiles. Furthermore binders are insulators that degrade ink conductivity. To achieve both low temperature processing and high conductivity, we’ve developed a binder-free strategy combining with rolling compression to enhance printed graphene conductivity^34^. Figure 1 shows how to make high conductivity printed graphene, combining with inserts of optical microscope (OM) and scanning electron microscope (SEM) photos of the sample, both above views and cross sectional views. As seen in Fig. 1, the conductive ink Gra-ink 102E (BGT Materials Ltd), containing graphene nanoflakes, dispersants and solvents, is coated on substrate. The OM photo with 1000 × magnification of the ink coating is shown in Fig. 1(a) and the graphene nanoflake suspensions can be observed. After drying process at 100 °C for 10 minutes, solvents are volatilized, and graphene nanoflakes coating is left on the substrate. It should be mentioned that, this low drying temperature is compatible with substrates like papers and/textiles. Even without binders, the free-standing graphene coating is robust and flexible, and their excellent film-forming ability makes the nanoflakes adhesive on substrate^35^. However, the graphene coating at this stage is highly porous, as illustrated in Fig. 1(b), leading to high contact resistance and unsmooth pathways for electron transport. To enhance the conductivity, a rolling compression is adopted to improve the adhesion of graphene nanoflakes. After compressing procedure, the graphene coating becomes highly dense and the printed graphene forms, as seen in Fig. 1(c). To make the compression process more visible, cross sectional SEM images of four samples with different compression ratios are given in Fig. 1(e–h). Figure 1(e) shows the uncompressed case, and Fig. 1(h) is of highest compression ratio 81%. To have better observation and fit the scope, magnifications of 500 × , 1000 × , 2000 × , 3000 × , are used for samples in Fig. 1(e–h), respectively. Obviously from Fig. 1(e–h), with the increase of compression ratio, graphene laminate thickness decreases.

The conductivity and surface resistance of the printed graphene under various compression ratios (the compression ratio is defined as the ratio of the thickness decrement of compressed sample over un-compressed sample thickness) are measured, as shown in Fig. 1(d). It can be seen, when compression ratio is 0%, namely graphene coating without compression, the thickness is 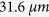 and the conductivity is 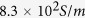. Its sheet resistance 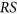 is calculated to be 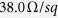 with 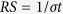. With the increase of compression ratio, the conductivity rises and sheet resistance decreases accordingly. When the compression ratio is 81%, i.e., the thickness of the printed graphene is 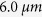, the conductivity increases to 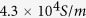, which means the conductivity is improved more than 50 times. Also, the sheet resistance is decreased to 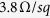, one tenth of un-compressed sample.

### Printed graphene enabled flexible transmission lines

TLs are basic structures designated to carry signals and are essential for RF circuits, or indeed any electronic cirucits^36^. As a proof of concept, we have designed and characterized some simple printed graphene enabled TL structures to investigate their feasibility for RF signal transmission.

**Figure 2:**
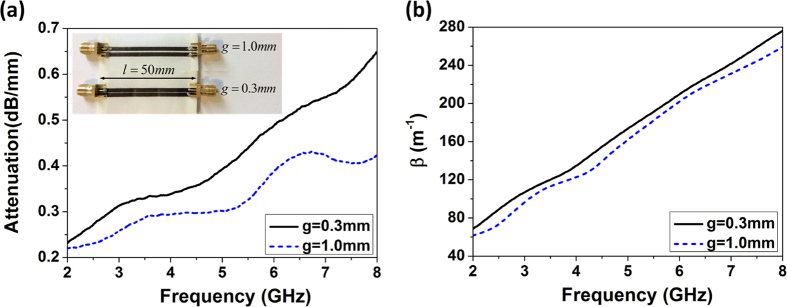
Performances of the transmission lines with various line gaps. (**a**) Attenuation of the transmission lines, and the insert is two transmission line samples with different line gaps, 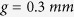 and 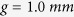, respectively, and (**b**) Phase constants *β* of the transmission lines.

The performance of a TL is mainly determined by material and geometrical parameters such as material losses, substrate material dielectric constant, line gaps, signal line thickness and etc. The insert in Fig. 2(a) shows two samples of TLs with different gaps between the lines. As it can be seen, a SMA connector is connected at each port of the line using conductive epoxy. The length of the lines is 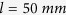, and the gaps are 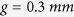 and 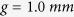, respectively.

The scattering parameters of these lines are measured using Agilent E5071B VNA (See Supplementary Fig. S1) and propagation constant can be calculated using the following equations^37^,


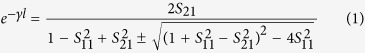



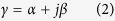


where *α* and *β* are attenuation constant and phase constant, respectively. To eliminate the effect of impedance mismatch on analyzing conductor loss, absorption attenuation, which is defined as the ratio of power entered into the input port of the network over the output power of the network, is calculated by^38^.


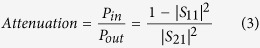


The attenuation is unitized to per 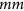 and displayed in Fig. 2(a). It can be seen that the wider the line gap, the lower the attenuation. This is because the electromagnetic field is concentrated mainly at inside edges of the lines; smaller gap makes the field more intensive, thus causes more conductor loss. However, it’s worthy to point out that the line gap cannot be set arbitrarily as it determines the characteristic impedance of the TL. As expected, the attenuation increases with frequency. The relatively high attenuation in these TLs is due to the thin thickness of the printed graphene. Thickness of the printed graphene in this report is 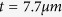
with conductivity 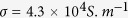. Its skin depths, from 2 GHz to 8 GHz, are between 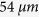 to 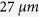, which means the printed graphene thickness is only 14.3% to 28.5% of its skin depth. To reduce attenuation in practical applications, normally conductor thickness should be 3–5 times of its skin depth. Increasing the printed graphene thickness is an effective way to obtain lower attenuation. Besides, from Fig. 2(b), the propagation constant is almost linear with frequency, revealing that there is little phase distortion in the printed graphene TLs, which is desirable in practical RF applications.

**Figure 3:**
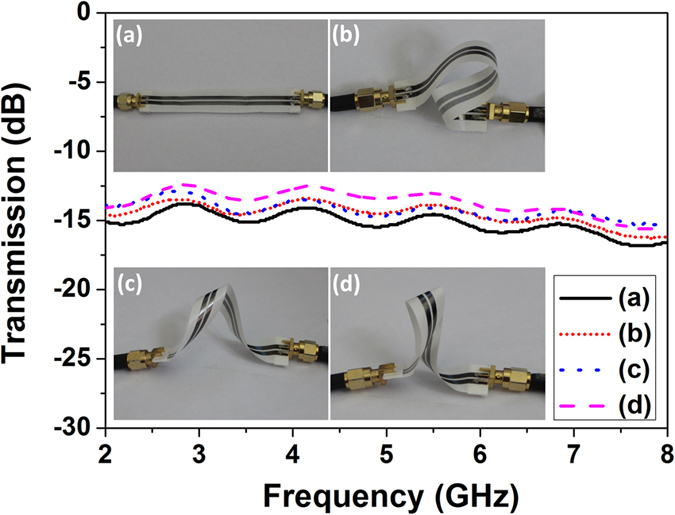
Un-bended, bended and twisted transmission lines and their transmission performances.

Moreover, the superior flexibility of the printed graphene enabled TLs is experimentally verified with the lines of 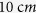 length and 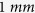 gap, as shown in Fig. 3. Four cases were examined. The printed graphene TL was not bended in Fig. 3(a), bended in Fig. 3(b) but not twisted, bended and twisted in Fig. 3(c,d). It is clearly evident that the bending and twisting of the printed graphene TLs do not alter the transmission coefficients much, highly desirable for wearable applications. The slight differences between the four cases are caused by the mutual coupling between different segments of the TLs. For instance, the un-bended case has less transmission coefficient than other three cases because no mutual coupling happens between different parts of the line. TLs in Fig. 3(b,c) have less coupling than that in (d), as segments of the line in (d) are placed spatially closer and more mutual coupling is introduced. It should be pointed out that the TLs in Fig. 3 have not been optimized for impedance matching (see in Supplementary Fig. S2). Higher transmission coefficient can be achieved with better impedance matching. As expected, the transmission coefficients for all the cases decrease as frequency increases.

### RF/Microwave antennas for on-body wearable communications system

**Figure 4:**
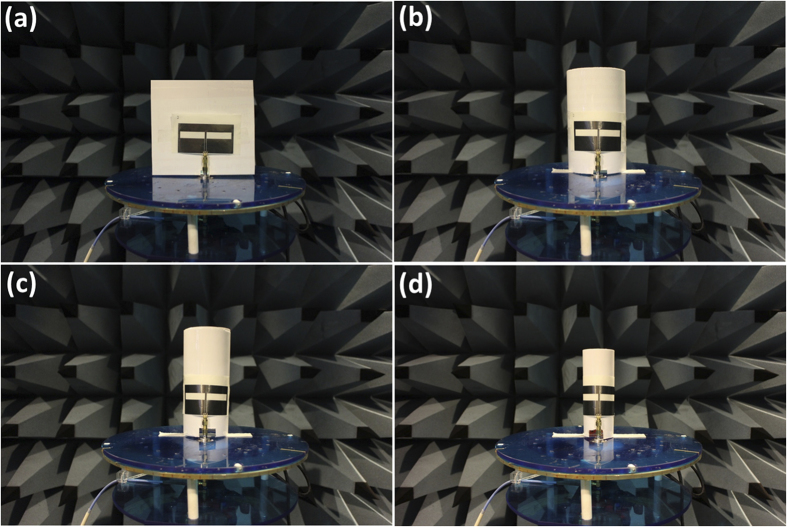
Printed graphene enabled antenna bended on cylinders with various radii, (a) un-bended, (b) bended with *r* = 5.0 *cm*, (c) bended with *r* = 3.5 *cm* and (d) bended with *r* = 2.5 *cm*.

Antenna is used to send and receive RF signals in communications systems. For wearable communications systems, both mechanical flexibility and effective radiation are demanded. For the first time, effective radiation of printed graphene enabled flexible and wearable antenna is experimentally demonstrated in communication frequency bands, such as mobile cellular and WiFi spectrums. Figure 4 shows the same printed graphene antenna bended and pasted on cylinders of different radii for flexibility and conformability tests. Figure 4(a) illustrates the un-bended antenna and (b), (c) and (d) show the antenna attached on cylinders with radius of 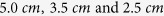, respectively. The antenna is a typical CPW fed slot antenna and printed on paper. The antenna’s parameters can be found in Supplementary Fig. S3.

**Figure 5:**
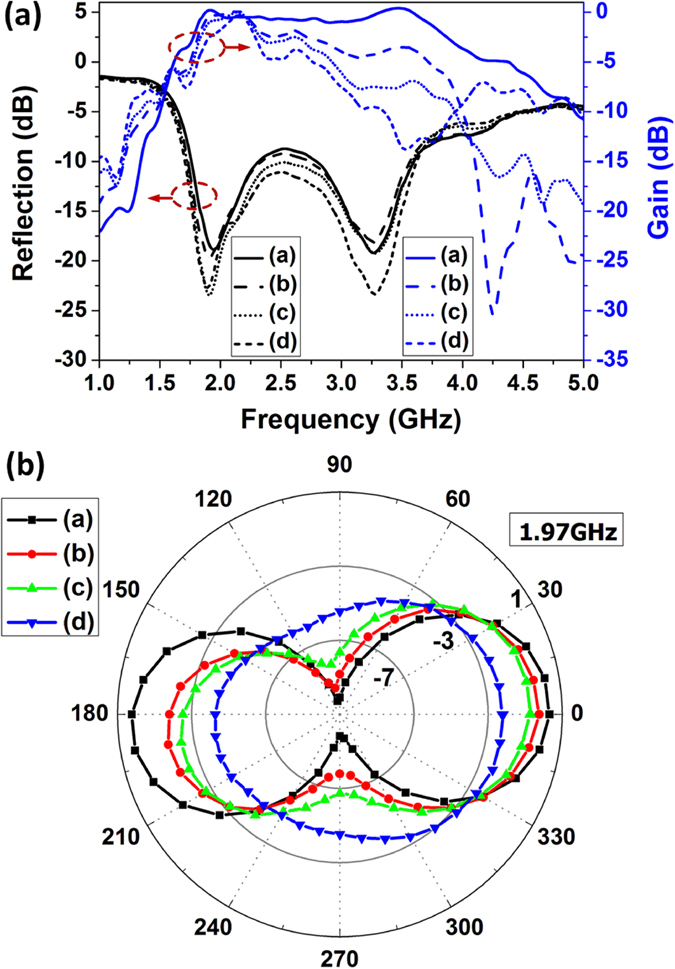
Measured results of the printed graphene enabled antenna bended on cylinders with different radii, as shown in Fig. 4; Accordingly, curves (a–d) correspond to un-bend, bended with radius of 5.0 *cm*, 3.5 *cm* and 2.5 *cm*, respectively. (**a**) Reflection coefficients and realized gains and (**b**) Radiation patterns at 1.97 GHz.

The reflection coefficients of the antenna under these four different bending cases were measured using VNA (Agilent E5071B), and the gain was obtained using three-antenna method^39^, displayed together in Fig. 5(a). It can be seen that when the antenna is un-bended the reflection coefficient S_11_ at 1.97 GHz is −18.7 dB, and another peak is at 3.26 GHz with −19.2 dB, revealing good impedance matching. The reflection coefficient is under −8 dB from 1.73 GHz to 3.77 GHz, which covers the bands for Wi-Fi, Bluetooth, WLAN (wireless local area network) and mobile cellular communications. The maximum gain is 0.2 dBi at 1.92 GHz and above −1 dBi from 1.82 GHz to 3.72 GHz, demonstrating an effective radiation from the printed graphene antenna to the free space. With comparison of reflection coefficients corresponding to different bending cases, it can be seen that the reflection coefficients are not sensitive to the bending and do not vary much. The impedance matching points are almost unchanged. However, the antenna gain changes, especially at higher frequency region. This is because the antenna gain is determined by current distribution on the antenna. When the antenna is bended, the current distribution will be altered, leading to variation on antenna gain performances. Despite that gain at higher frequency band around 3.26 GHz degrades visibly with increasing bending, the gain at lower band around 1.9 GHz to 2.2 GHz has much less variations. This frequency band is where wireless wearable communications systems operate. The experimental data here demonstrate that even when the printed graphene antenna is bended, the radiation at this this frequency band can still be effective.

The corresponded radiation patterns under cases (a)–(d) at 1.97 GHz in elevation plane were also measured using antenna measurement system (Antenna Measurement Studio 5.5, Diamond Engineering). The data were recorded for every 10 degree rotation and shown in Fig. 5(b). From the radiation patterns, it can be seen that cases (a)–(c) are quite similar despite of minor decrease of maximum gain. Pattern of case (d) is rather different from the other three because the much severely bended antenna poses much alteration in current distribution and causes the antenna’s resonant frequency to shift. It is found that in this case the resonant frequency has shifted to 2.16 GHz. The radiation pattern at 2.16 GHz can be found in Supplementary Fig. S4.

**Figure 6:**
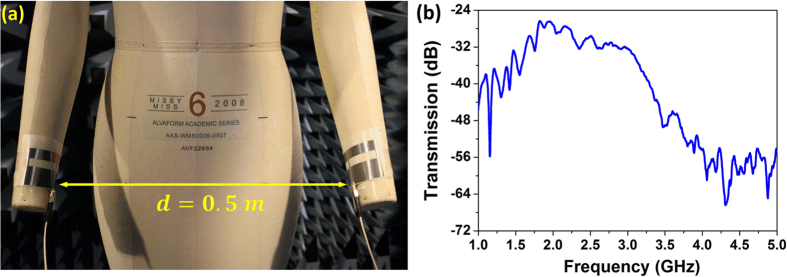
Measurement of transmission between two on-body printed graphene enabled wearable antennas. (**a**) Measurement setting of the wearable antennas on mannequin and (**b**) Transmission between two antennas attached on hands of mannequin with 0.5 m separation.

With the aforementioned verification for the flexibility and efficient radiation of the printed graphene enabled antenna, here we go a step further to prove its potentials in wireless wearable communications systems by presenting a real life scenario shown in Fig. 6(a). It depicts an on-body communications testing setup. On-body communications is signal transmitting/receiving between on-body networks and systems^10^,^40^. In this setup, the graphene antennas are bended and attached on mannequin’s hands to transmit/receive RF signals. The transmission coefficient between the two antennas is shown in Fig. 6(b). When the distance between the two antennas is 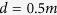, the transmission coefficient from 1.67 GHz to 2.87 GHz is above −32 dB, which is more than 20 dB higher than −55 dB observed out of band above 3.8 GHz. The measured results verifying that RF signal can be effectively radiated and received by these two graphene antennas.

## Discussion

We have presented highly conductive and flexible printed graphene TLs and antennas using graphene nanoflakes aiming for wireless wearable communications applications. The feasibility of using printed graphene to transmit/receive RF signals through wires and wirelessly has been demonstrated experimentally. The superior flexibility of the printed graphene enabled TLs and antennas has been fully verified with measurements under different bending and twisting cases. An on-body signal transmission on mannequin has been also presented by using printed graphene antennas conformed to the model’s arms for radiating and receiving RF signals wirelessly. It is clearly evident that the sound mechanical flexibility and effective radiation of the printed graphene enabled antennas have successfully facilitated the on-body wireless communications. This work has unambiguously proved that printed graphene can bring transformative change to the formation of RF passive components such as TLs and antennas for wearable applications. Furthermore, the fabrication process is simple and low cost and thus suitable for commercial mass production. Combined with other profound advantages in lightweight, mechanical flexibility and environmental friendliness, printed graphene can be ideal for low cost consumable wearable electronics.

## Methods

### Preparation of samples

Graphene nanoflakes were dispersed in N-Methyl-2-pyrrolidone (NMP) solvent to form a uniform 10 wt% graphene ink, i.e. Grat-Ink 102E. Less than 1 wt % of non-ionic polymer-type surfactants was contained in the ink. The presence of the surfactants improve the dispersion of graphene flakes and viscosity helping in deposition of a uniform film. The Grat-Ink 102E, prepared as described above, was used to print the samples in this study. Conventional paper was used as the substrate, and the patterns of the samples were printed by 150 mesh stainless-steel screen via a manual laboratory screen-printing table. They were dried at 100 °C for 10 minutes. Further a rolling compression procedure was followed using compression roller (SERP02, Shining Energy, Taiwan) to obtain highly dense graphene laminates. The different compression ratio and thickness of samples are controlled by adjusting the space between two rollers. High reproducibility can be guaranteed when roller distance is fixed during operation. The sheet resistance of the graphene laminate pattern was measured by the 4-point probe (RM3000, Jandel, UK). The thicknesses of un-compressed and compressed graphene patterns were measured with digital thickness gauge (PC-485, Teclock, Japan). A total of 10 measurements at different spots were carried out to obtain the average value of each sample.

## Additional Information

**How to cite this article**: Huang, X. *et al.* Highly Flexible and Conductive Printed Graphene for Wireless Wearable Communications Applications. *Sci. Rep.* **5**, 18298; doi: 10.1038/srep18298 (2015).

## Supplementary Material

Supplementary Information
