# Supplementary material for: Highly Flexible and Conductive Printed Graphene for Wireless Wearable Communications Applications
Source: Sci Rep. 2015 Dec 17;5:18298. doi: 10.1038/srep18298 (PMC4682142; doi:10.1038/srep18298)
Supplement: Supplementary Information [file srep18298-s1.pdf]

Highly Flexible and Conductive Printed Graphene for Wireless Wearable Communications Applications (Supplementary Information)

Xianjun Huang,[1] Ting Leng,[1] Mengjian Zhu,[2] Xiao Zhang,[1] JiaCing Chen,[3] KuoHsin Chang,[3] Mohammed Aqeeli,[1] Andre K. Geim,[4] Kostya S. Novoselov,[2] Zhirun Hu,[1,*]

[*]Correspondence: Zhirun Hu (z.hu@manchester.ac.uk)

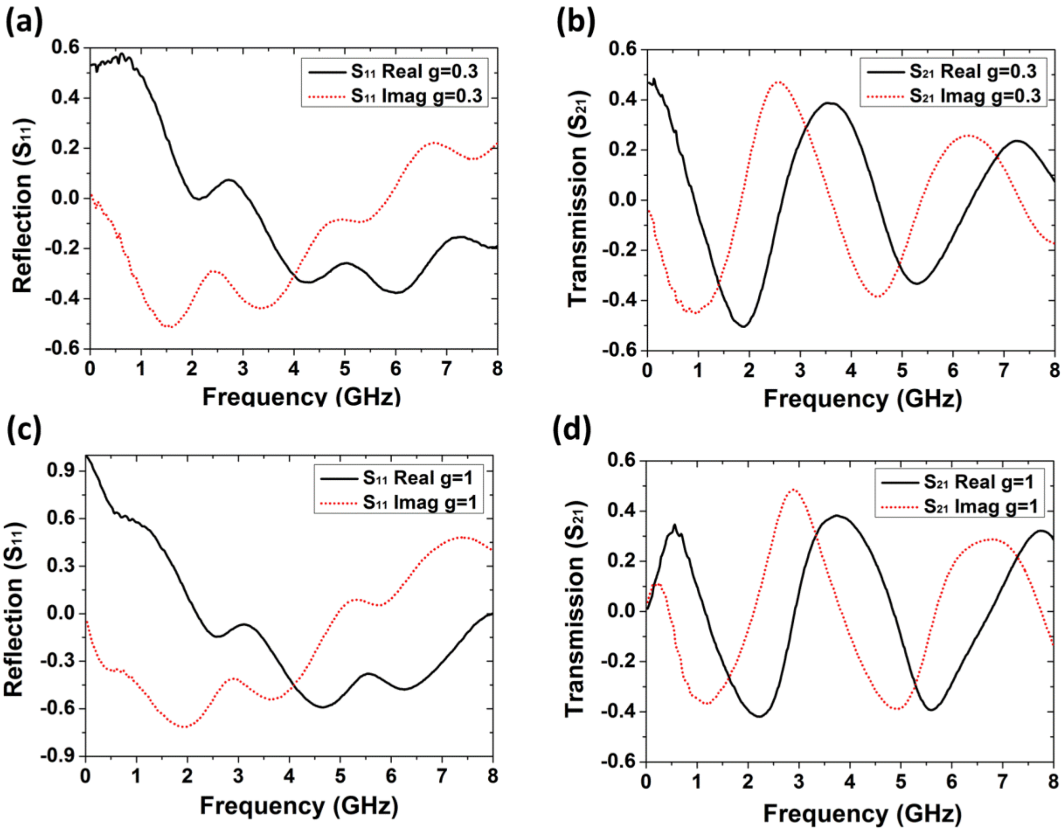

Supplementary Figure S1 Measured S parameters of slot transmission line. (a) Real and imaginary part of S_{11} when g=0.3mm, (b) Real and imaginary part of S_{21} when g=0.3mm, (c) Real and imaginary part of S_{11} when g=1mm, (b) Real and imaginary part of S_{21} when g=1mm.

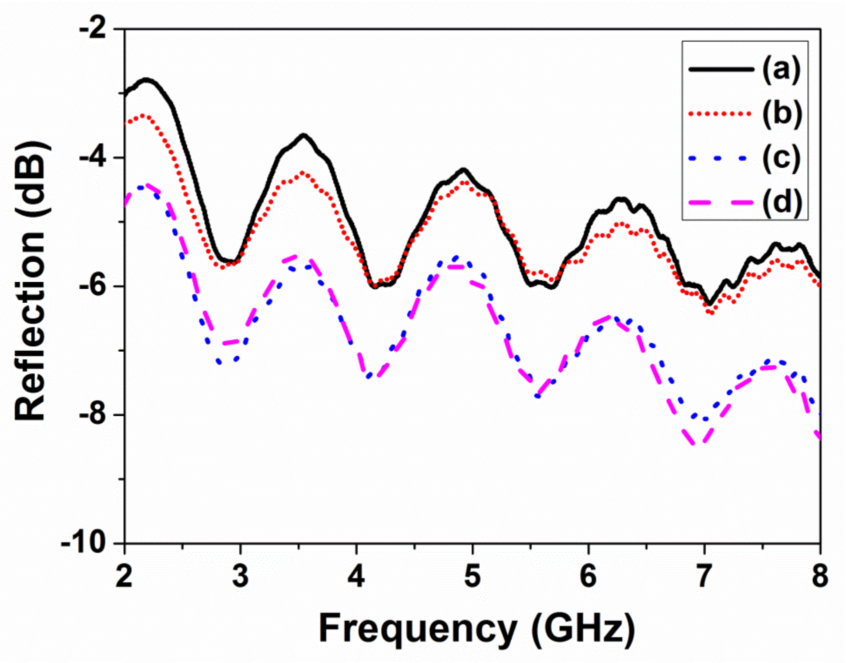

Supplementary Figure S2 Reflections of Un-bended (a), bended (b, c) and twisted (d) slot lines.

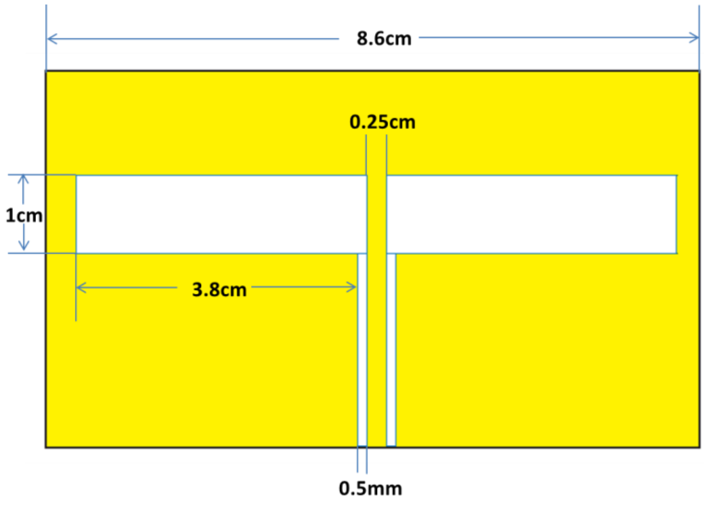

Supplementary Figure S3 CWP fed slot antenna layout parameters

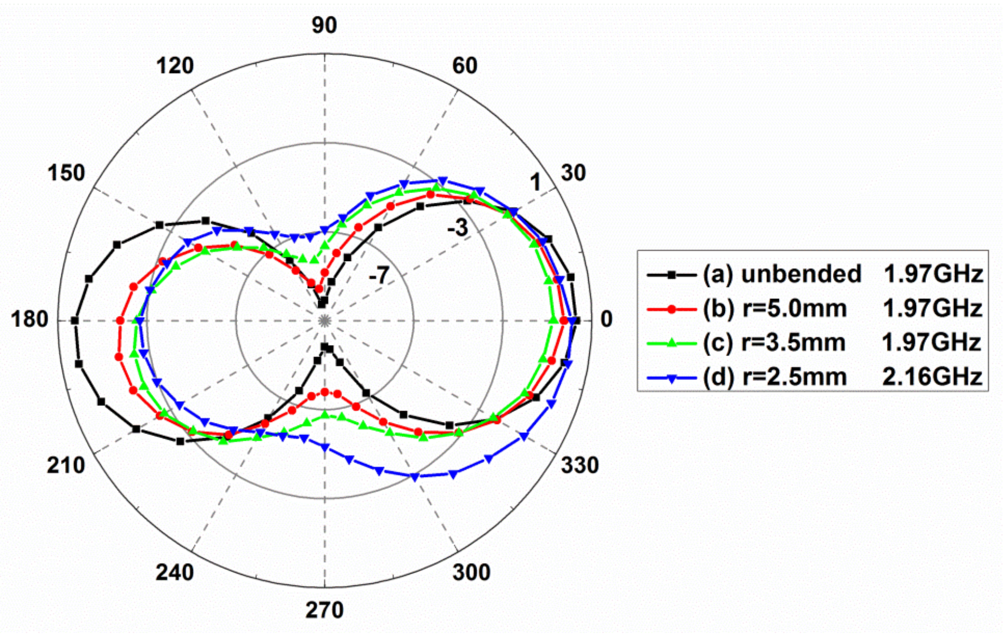

Supplementary Figure S4 Comparison of measured radiation pattern at 1.97GHz of case (a)-(c), and radiation pattern at 2.16GHz of case (d). It can be seen that in case (d) $r = 2.5cm$, at 2.16GHz, the maximum gain frequency point, the radiation pattern similar with other cases (a)-(c).

Reference

[1] Nithisopa, K., Nakasuwan, J., Songthanapitak, N., Anantrasirichai, N., & Wakabayashi, T. (2007). Design CPW fed slot antenna for wideband applications.
